# Effects of Platelet-Rich Plasma on Cellular Populations of the Central Nervous System: The Influence of Donor Age

**DOI:** 10.3390/ijms22041725

**Published:** 2021-02-09

**Authors:** Diego Delgado, Ane Miren Bilbao, Maider Beitia, Ane Garate, Pello Sánchez, Imanol González-Burguera, Amaia Isasti, Maider López De Jesús, Jone Zuazo-Ibarra, Alejandro Montilla, María Domercq, Estibaliz Capetillo-Zarate, Gontzal García del Caño, Joan Sallés, Carlos Matute, Mikel Sánchez

**Affiliations:** 1 Advanced Biological Therapy Unit, Hospital Vithas Vitoria, 01008 Vitoria-Gasteiz, Spain; diego.delgado@ucatrauma.com (D.D.); maider.beitia@ucatrauma.com (M.B.); ane.garate@ucatrauma.com (A.G.); pello.sanchez@ucatrauma.com (P.S.); 2 Arthroscopic Surgery Unit, Hospital Vithas Vitoria, 01008 Vitoria-Gasteiz, Spain; anemiren.bilbao@ucatrauma.com; 3 Department of Neurosciences, Faculty of Pharmacy, University of the Basque Country (UPV/EHU), 01008 Vitoria-Gasteiz, Spain; imanol.gonzalezb@ehu.eus (I.G.-B.); gontzal.garcia@ehu.eus (G.G.d.C.); 4 Bioaraba, Neurofarmacología Celular y Molecular, 01008 Vitoria-Gasteiz, Spain; amaia.isasti@ehu.eus (A.I.); maider.lopez@ehu.eus (M.L.D.J.); joan.salles@ehu.eus (J.S.); 5 Department of Pharmacology, Faculty of Pharmacy, University of the Basque Country (UPV/EHU), 01008 Vitoria-Gasteiz, Spain; 6 Centro de Investigación Biomédica en Red de Salud Mental (CIBERSAM), 28029 Madrid, Spain; 7 Achucarro Basque Center for Neuroscience, CIBERNED and Departamento de Neurociencias, Universidad del País Vasco (UPV/EHU), 48940 Leioa, Spain; jone.zuazo@ehu.eus (J.Z.-I.); alejandro.montilla@ehu.eus (A.M.); maria.domercq@ehu.eus (M.D.); estibaliz.capetillo@ehu.eus (E.C.-Z.); carlos.matute@ehu.eus (C.M.); 8 IKERBASQUE, Basque Foundation for Science, 48009 Bilbao, Spain

**Keywords:** platelet-rich plasma, growth factors, aging, neurons, microglia, central nervous system

## Abstract

Platelet-rich plasma (PRP) is a biologic therapy that promotes healing responses across multiple medical fields, including the central nervous system (CNS). The efficacy of this therapy depends on several factors such as the donor’s health status and age. This work aims to prove the effect of PRP on cellular models of the CNS, considering the differences between PRP from young and elderly donors. Two different PRP pools were prepared from donors 65–85 and 20–25 years old. The cellular and molecular composition of both PRPs were analyzed. Subsequently, the cellular response was evaluated in CNS in vitro models, studying proliferation, neurogenesis, synaptogenesis, and inflammation. While no differences in the cellular composition of PRPs were found, the molecular composition of the Young PRP showed lower levels of inflammatory molecules such as CCL-11, as well as the presence of other factors not found in Aged PRP (GDF-11). Although both PRPs had effects in terms of reducing neural progenitor cell apoptosis, stabilizing neuronal synapses, and decreasing inflammation in the microglia, the effect of the Young PRP was more pronounced. In conclusion, the molecular composition of the PRP, conditioned by the age of the donors, affects the magnitude of the biological response.

## 1. Introduction

Platelet-rich plasma (PRP) is a biologic therapy that uses patients’ own blood to obtain products with a higher platelet concentration than in circulation levels. Blood centrifugation usually aims to separate its components in order to discard substances such as red blood cells, while concentrating therapeutic elements like cytokines and growth factors. Although there are several methods to obtain PRP products, all of them have in common a biological basis, which relies on the release of therapeutic molecules stored in platelets [1]. These growth factors, together with other plasmatic biomolecules, have been found to promote natural healing responses across multiple medical fields such as dentistry, dermatology, gynecology, urology, sports medicine, and orthopedics, including peripheral nerves [2], and there has been increasing interest in the central nervous system (CNS) [3].

The action of PRP in the CNS is evidenced by several works that demonstrate the therapeutic potential of PRP products as neuroprotective, neurogenic, and neuroinflammatory modulators [4,5,6,7,8]. Indeed, some growth factors present in PRP preparations have been described as key regulators of diverse processes such as proliferation, migration, fate choice, survival, and cell differentiation [2]. In vitro experiments carried out on neuronal cultures from an Alzheimer’s disease mouse model demonstrated that PRP could reduce the neurotoxicity induced by aggregated β-amyloid in primary neuronal cultures. Moreover, the living cell number after treatment with PRP increased, and chronic intranasal administration of PRP in an Alzheimer’s disease mouse model generated neuroprotection [4]. In a mouse model of Parkinson’s disease, the same research group observed a reduction in neuroinflammatory processes mediated by microglia, using PRP as treatment [5].

However, the efficacy potential of this autologous cocktail could depend on several factors such as the donor’s health status or age, being key factors in this kind of autologous system. Previous studies have shown that the cytokines and biomolecules of elder and young people present differences [9]. These differences may have consequences on the results obtained with treatments based on this technique due to molecular changes in PRP. In fact, diverse studies showed that Aged PRP contains more proinflammatory molecules than Young PRP [10].

The aim of this work is to prove the effect of PRP on cellular models of the CNS, considering the differences between PRP from young and aged donors. For that purpose, an in vitro study on neuronal progenitor cells was carried out first, evaluating cell proliferation and neurogenesis. After that, rat brain cell populations were employed in vitro to analyze the effect of both PRP on neuronal synapses and microglia inflammation.

## 2. Results

### 2.1. PRP Characterization: Growth Factors and Cytokine Measurement

**Figure 1:**
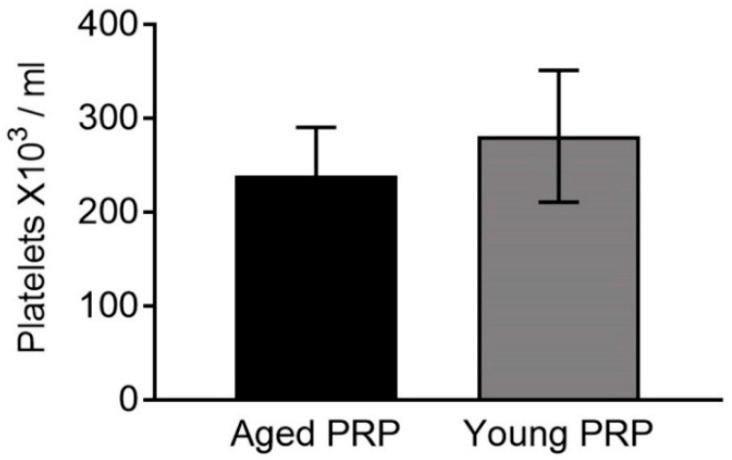
Platelet concentration of the two platelet-rich plasma (PRP) groups. Error bars = standard deviation.

The mean age of Aged PRP and Young PRP was 73.71 ± 5.00 and 22.13 ± 1.81 years, respectively (Table 1). According to the latest coding system and minimum reporting requirements for PRP studies, the type of PRP used in this study was 12-00-11 and the characteristics of both PRPs are reported in Table 2 [11]. The PRP employed to elaborate Aged and Young pools did not contain leukocytes, and there were no significant differences in the platelet number (Figure 1).

**Figure 2:**
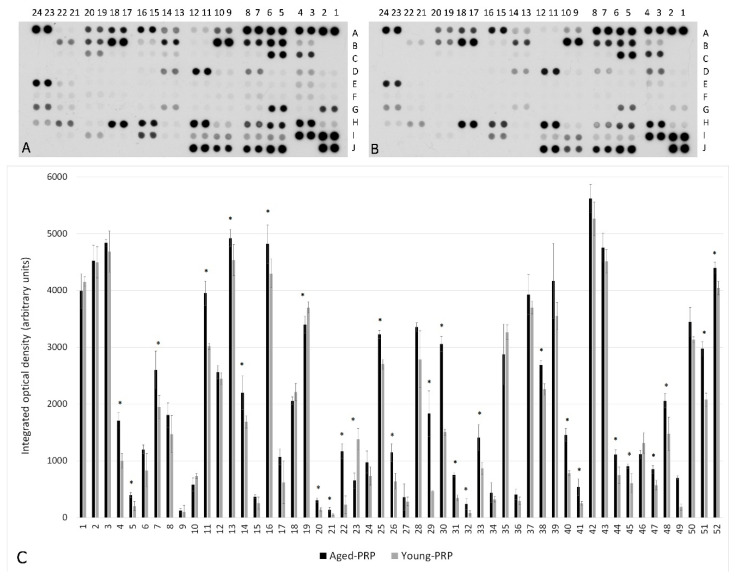
Molecular analysis of Young and Aged PRPs. Cytokine array of Aged PRP (**A**). Cytokine array of Young PRP (**B**). Molecular levels in the Aged PRP with respect to the Young PRP (**C**). The numbers and letter codes of panels A and B make reference to the coordinates of Table 4. The numbers of the X axis in panel C make reference to the number of each molecule in Table 4. * *p* < 0.05. Error bars = standard deviation.

However, after cytokine and growth factor content analysis, some differences between the PRPs were detected: the Young PRP group presented higher levels of growth differentiation factor (GDF-11) (only detected in young plasma), insulin-like growth factor (IGF) (*p* < 0.001), platelet-derived growth factor (PDGF) (*p* = 0.016), and transforming growth factor-β (TGF-β) (*p* = 0.025). The Aged PRP group showed higher levels of C-C motif chemokine 11 (CCL-11) (*p* = 0.022) and hepatocyte growth factor (HGF) (*p* = 0.001) (Table 3). In addition, PRP from aged donors had a more inflammatory profile, with higher pro-inflammatory cytokine levels than the PRP from young donors (Table 4 and Figure 2).

### 2.2. Effect of PRP on BrdU Incorporation in NT2 Progenitors

**Figure 3:**
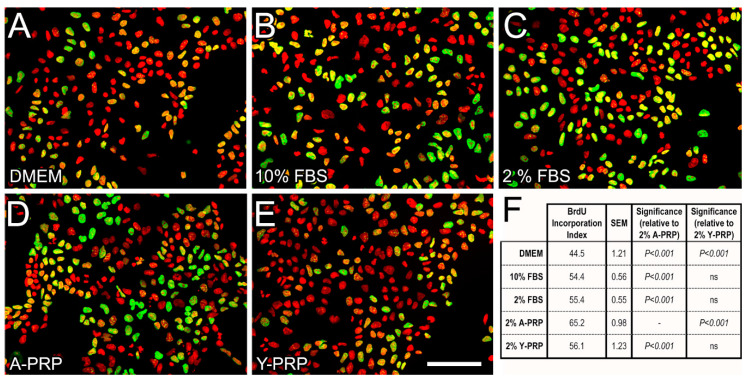
Epifluorescence microscopy images of NT2 cells showing the effect of replacement of FBS by PRP on BrdU incorporation. (**A**–**E**). Epifluorescence microscopy images of NT2 cells subjected to a 4-h BrdU pulse after growing for 24 h in either serum-free DMEM medium (**A**) or in 10% FBS (**B**), 2% FBS (**C**), 2% Aged-PRP supplemented DMEM (**D**), or 2% Young-PRP supplemented DMEM (**E**). Cells were processed for double immunofluorescence against BrdU (green), combined with Hoechst’s chromatin staining (pseudocolored red). Scale bar: 50 μm (**F**) Table showing results of BrdU incorporation analysis.

BrdU incorporation was analyzed in serum-starved NT2 progenitor cells after stimulation for 24 h with either FBS (2% or 10%) or PRP (2% Aged PRP or 2% Young PRP) (Figure 3A–E). As predicted, a significant increase in BrdU Incorporation Index (*p* < 0.001, one-way ANOVA and Bonferroni post hoc test) was observed in all stimulation conditions compared to unstimulated cells (kept in serum-free DMEM). Treatment with 2% Aged PRP resulted in a significant increase in BrdU Incorporation index, compared with cells stimulated with 2% FBS or 2% Young PRP. Moreover, 2% Aged PRP led to a higher BrdU incorporation index compared to 10% FBS (Figure 3F), indicating that Aged PRP increases mitotic activity in NT2 progenitors.

### 2.3. Effect of PRP on Cell Survival and Morphology of AraC-Differentiated NT2 Cells

**Figure 4:**
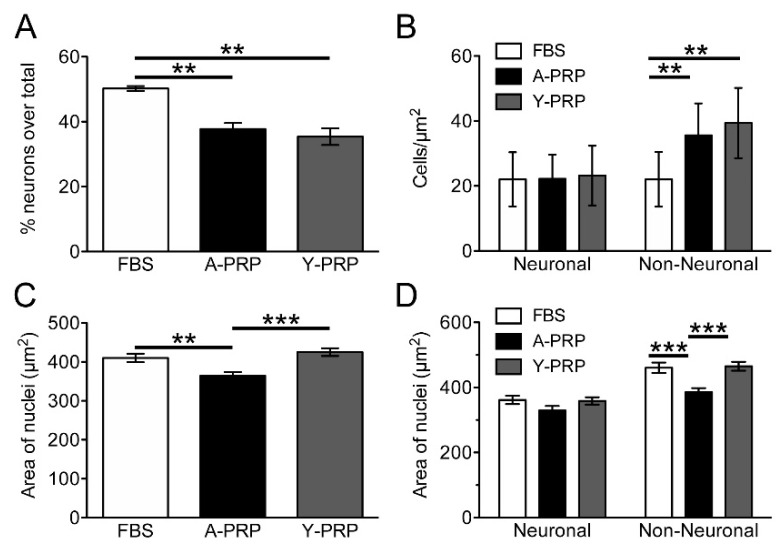
Analysis of phenotype density and area of cell nuclei in cultures of AraC-treated NT2 cells subjected to fetal bovine serum (FBS), Aged PRP, or Young PRP treatment. Percentage of cell of neuronal phenotype (**A**). Density of neuronal and non-neuronal cells (**B**). Area of cell nuclei in the whole cell population (**C**). Area of nuclei of neuronal and non-neuronal cells (**D**). ** *p* < 0.01, *** *p* < 0.001. Error bars = standard deviation. A-PRP: Aged PRP; Y-PRP: Young PRP.

AraC-differentiated NT2 cells were treated with Aged PRP and Young PRP for 72 h. The analysis of cell density showed that either treatment led to a decrease in the percentage of neuronal cells (Figure 4A), which was due to an increase in the cell survival of the non-neuronal population, as no changes in the density of neuronal NT2N cells were observed, whereas the density of non-neuronal cells was significantly higher after treatment with either Aged PRP or Young PRP (Figure 4B). With regard to neuronal morphology, treatment with Aged PRP, but not with Young PRP, produced a decrease in the mean area of the cell nucleus (Figure 4C). A separate analysis of the NT2N and non-neuronal populations showed that this change mainly occurred in non-neuronal cells (Figure 4D). No differences were found between untreated and PRP-treated NT2N cells in terms of the number of neurites per cell or the total length of the neuritic tree, despite the slight decrease in the average number of neurites after treatment with Young PRP (FBS, 2.28 ± 0.12 SEM, *n* = 112; Aged PRP, 2.21 ± 0.14 SEM, *n* = 112; Young PRP, 2.07 ± 0.12, *n* = 118) and in the total length of the neuritic tree after treatment with either Aged PRP or Young PRP (FBS, 139.8 µm ± 8.5 SEM, *n* = 112; Aged PRP, 127.4 µm ± 7.4 SEM, *n* = 112; Young PRP, 127.2 µm ± 6.7, *n* = 118).

### 2.4. Effect of PRP on Toxicity and Quantification of Pre- and Postsynaptic Markers

**Figure 5:**
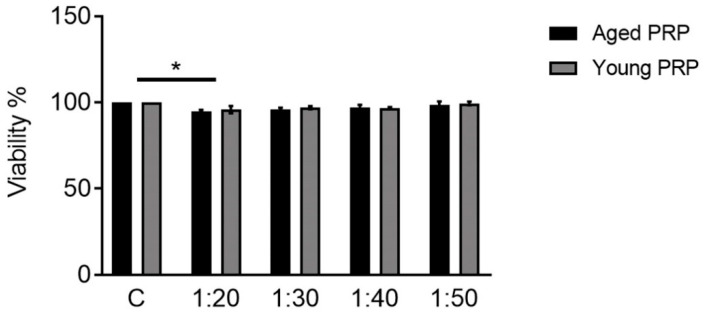
Evaluation of PRP toxicity with calcein assay in primary cultures of neurons from rat E16-18 cortices, with different plasma concentrations (dilutions): 1:20, 1:30, 1:40, or 1:50. * *p* < 0.05. Error bars = standard deviation.

In order to analyze the toxicity caused by the two PRPs, neuronal primary cultures from rat E16-18 cortices were cultured under both conditions and cellular viability was measured. Both Aged and Young PRP treatments did not show any significant toxicity or dilution ratios higher than 1:20 (Figure 5). Considering these results and in order to keep similarity in progenitor cell lines, the following assays were carried out with 2% Aged and Young PRPs.

**Figure 6:**
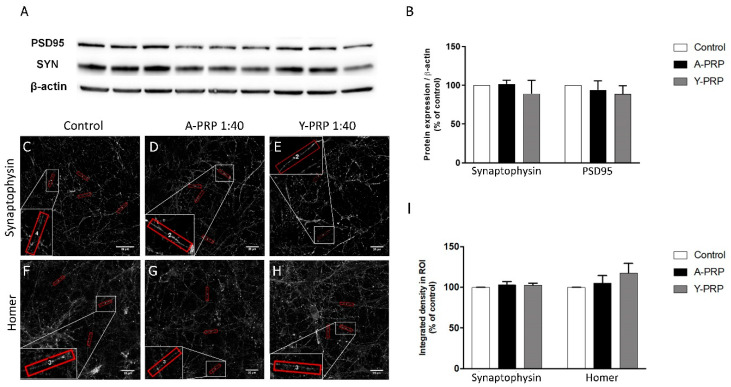
Synaptic protein expression. Protein expression of presynaptic (Synaptophysin) and postsynaptic (PSD-95) markers in neuronal cells treated in triplicate with control, Aged PRP, or Young PRP (**A**) and its quantification relative to the control treatment (**B**). (**C**–**H**) Immunohistochemical analysis of presynaptic (synaptophysin) and postsynaptic (Homer) markers in neuronal cells and its quantification by the integrated density in ROI relative to the control treatment (**I**). A-PRP: Aged PRP; Y-PRP: Young PRP. Error bars = standard deviation.

**Figure 7:**
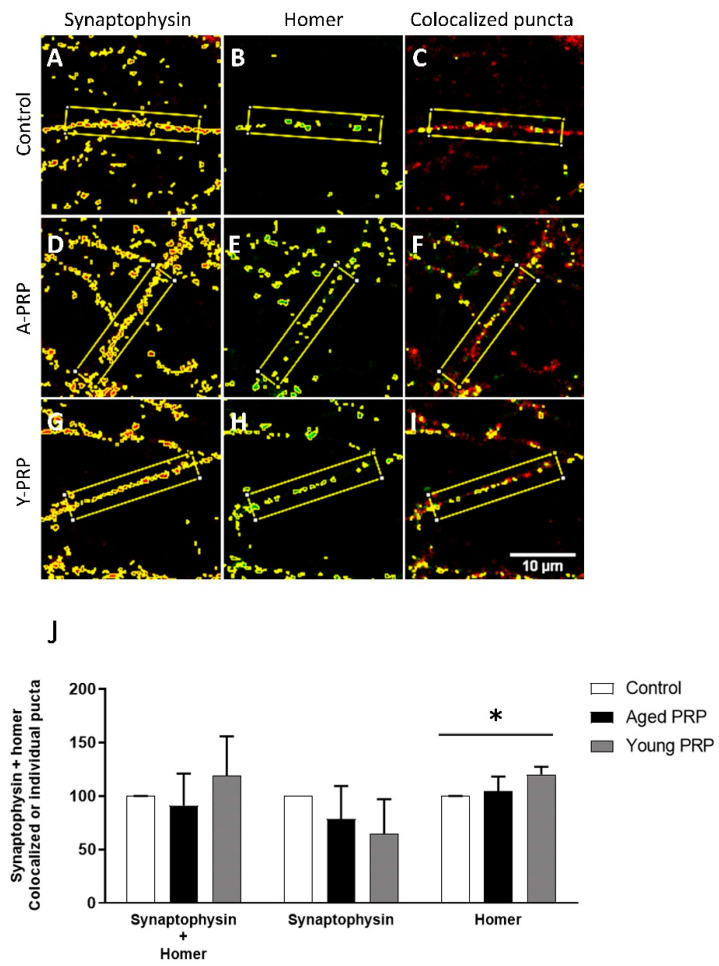
Young PRP increases Homer postsynaptic puncta. Representative images of individual Synaptophysin and Homer puncta and colocalized puncta in neuronal primary cultures treated with either Aged PRP or Young PRP (**A**–**I**). Quantification of individual Synaptophysin and Homer puncta and the colocalized Synaptophysin and Homer puncta (**J**). A-PRP: Aged PRP; Y-PRP: Young PRP. * *p* < 0.05. Error bars = standard deviation.

Once the concentration of the PRPs was stablished, both the expression of synaptic proteins and the number of synaptic puncta were quantified to evaluate the effect of the PRPs on synapses. As a first approach, expression of presynaptic marker Synaptophysin and postsynaptic maker PSD-95 was quantified in whole-cell lysates by Western blot (Figure 6A). Synaptophysin is a presynaptic protein involved in recycling of synaptic vesicles and PSD-95 is a postsynaptic density protein. This analysis revealed that PRP did not modify the global levels of those markers (Figure 6B). For a more detailed analysis, the expression of both pre- and postsynaptic markers was quantified at the neuritic level by immunofluorescence. For this analysis, in addition to the presynaptic marker Synaptophysin, the postsynaptic marker Homer was used instead of PSD-95 (Figure 6C–H). Homer, as PSD-95, is a postsynaptic density scaffolding protein and it is involved in the stability of the spines [12]. Although no significant differences in the intensity of the presynaptic marker Synaptophysin and postsynaptic marker Homer were observed in the presence of both Young and Aged PRPs, Homer expression showed a trend to increase (Figure 6I). Therefore, we decided to evaluate whether this trend could represent an effect on the number of synaptic puncta in the presence of PRPs. For this analysis, Synaptophysin and Homer were used. Interestingly, quantification of the pre- and postsynaptic puncta (Figure 7A–I), reveals a trend of increase in synaptic puncta represented by colocalization of Synaptophysin and Homer. Moreover, a significant increase in Homer-positive postsynaptic puncta was observed (Figure 7J).

### 2.5. Effect of PRP on Microglia Inflammation

**Figure 8:**
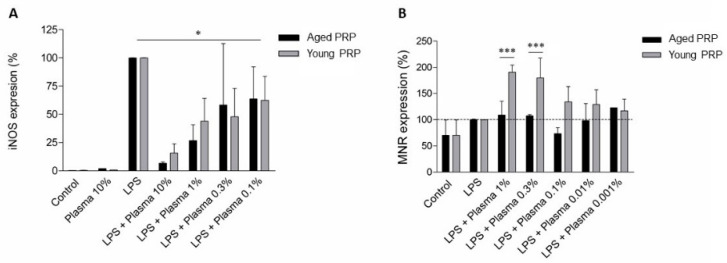
Expression of enzyme-inducible nitric oxide synthase (iNOS), a pro-inflammatory marker indicative of the M1 inflammatory phenotype (**A**) and mannose receptor (MNR), an anti-inflammatory marker indicative of the M2 restorative phenotype (**B**) in microglia of rats treated with different plasma concentrations. * *p* < 0.05; *** *p* < 0.001. Error bars = standard deviation.

The effect of the two PRPs on inflammation was tested in primary cell cultures from the microglia of rat P0-2 cortices. Cells were activated with lipopolysaccharide (LPS) and interferon-gamma (IFN-γ) in order to cause inflammation, and the effect of both PRPs at different dilutions was compared. We analyzed the expression of a pro-inflammatory marker, inducible nitric oxide synthase (iNOS), and the expression of an anti-inflammatory marker, mannose receptor (MNR). Both PRPs had a potent anti-inflammatory effect since the lowest dose (0.1%) significantly inhibited iNOS expression. However, there were no differences in the inhibitory effect of both PRPs on the pro-inflammatory marker iNOS expression, as seen in the dose-response curve (Figure 8A). In addition, incubating the cells with Young PRP, but not with Aged PRP, increased the expression of the anti-inflammatory marker MNR (Figure 8B).

## 3. Discussion

There is growing interest in the use of PRP products in different medical applications, including in treatment for CNS pathologies, due to their neuroprotective, neurogenic, and neuroinflammatory properties. Several studies demonstrated the positive effects that PRP administration could produce in animal models of Parkinson’s or Alzheimer’s diseases [3,13]. However, since it is an autologous product, the effect of PRP may vary depending on the obtaining process, the type of PRP, or the characteristics of the donor.

In this sense, the donor’s age could be an important characteristic due to the differences that have been observed between plasma obtained from young and old populations [14,15]. The findings in this work showed differences between the PRP from young donors and aged donors on different biological processes involved in the CNS.

The processes of obtaining and types of PRP of the two products analyzed in this work were identical, with the unique variable being the age of the donors. Both PRPs presented a similar cellular composition, without differences in the number of platelets and without leukocytes or erythrocytes. On the contrary, the molecular composition was different, with variations in growth factors and in cytokines, with the PRP of elderly donors presenting higher levels of molecules inherent to aging, inflammation, and chronic diseases. These characteristics are in line with previous studies that demonstrated the influence of age on different levels of cytokines and growth factors [9,10].

The first processes analyzed were related to neuronal progenitor cells; considering these was a necessary preliminary step to the study of the neurons themselves. As a first approach, BrdU incorporation was assessed in NT2 progenitor cells in order to evaluate the proliferation process. Surprisingly, Aged PRP treatment, but not the Young pool, led to a significant increase in the BrdU incorporation index compared to control FBS, indicating a higher proliferation rate of neuronal progenitor cells. Although this result could suggest a better biological effect of Aged PRP, it may be due to the high pro-inflammatory cytokine content of Aged PRP. In fact, different works reported that neural progenitor cell proliferation might be altered and boosted in certain phases in an inflammatory environment [16,17,18], caused by the presence of higher levels of cytokines in Aged PRP than in the Young one.

Next, the neuronal differentiation of these progenitor cells was assessed by AraC treatment. Both PRPs caused a decrease in the percentage of neuronal cells due to an increase in non-neuronal ones. These data indicate that PRP did not contribute to neuronal differentiation of NT2 cells but increased the survival of progenitor ones, possibly avoiding the apoptosis produced by AraC treatment [19]. Contrarily, other authors reported PRP as a differentiation promoter in mesenchymal stem cells from bone marrow, adipose tissue, or dental apical papilla [20,21,22]. On the other hand, the capacity of PRP to abolish apoptosis was seen in other cell types such as chondrocytes [23], osteoblasts [24], and skeletal muscle cells [25]. Interestingly, progenitor neural cells treated with Young PRP showed a larger cell nucleus compared to those treated with Aged PRP, which could be indicative of processes that occur prior to cell differentiation [26,27].

After studying the processes in the neural progenitor cells, the next step was to evaluate the neuronal cells. In order to obtain a deeper evaluation of the effects of PRP on the central nervous system, primary cultures of neurons were employed. None of the PRPs modified the amount of the analyzed pre- and postsynaptic protein expression levels. However, Young PRP could play a role in the stability of the spine, since colocalization of pre- and postsynaptic puncta showed a trend of increase and Homer-positive postsynaptic puncta were significantly increased upon Young PRP treatment. Homer is a postsynaptic scaffold protein and has been reported to be involved, together with Shank protein, in the enlargement of the spine heads and promoting their stability [12]. It is difficult to pinpoint the greater therapeutic potential of Young PRP with respect to Aged PRP to a few molecules since the biological action of PRP is due to the synergistic action of its components. However, some molecules should be mentioned for their contribution to improving these biological processes. Thus, these results could be explained at least in part by the higher levels of CCL-11 found in Aged PRP, because CCL-11 increases with age and is involved in processes related to a decrease in neurogenesis. Parabiosis works carried out by Villeda et al., wherein old mice and young mice were surgically attached to share a circulatory system, demonstrated that CCL-11, among others, was a key molecule in brain Aged in the young mice joined to old mice [28,29]. Moreover, we must consider the presence of GDF-11 in the group of Young PRP but not in the Aged PRP, where the levels were lower than expected. This molecule could be involved with synaptic modulation [30] or with diseases related to age [31]. Furthermore, IGF was described as a key regulator of neurogenesis, helping to promote the proliferation and differentiation of neuronal cells [4].

Finally, the microglia are an important component of the central nervous system, essential to neurodevelopment and orchestrating the immune response and other processes such as synaptogenesis and neuron cell death [32,33]. As observed in the present study, Young, but not Aged PRP is able to increase the expression of the anti-inflammatory marker mannose receptor in microglia after LPS and IFN-gamma-induced inflammation. This suggests that the whole pro-inflammatory/anti-inflammatory balance of microglia activation varies depending on the age of the PRP donor and that Young PRP promotes a proregenerative phenotype in microglia. It was previously reported that PRP is able to activate microglia in a rat model of spinal cord injury [34]. Again, it is difficult to relate the improvements caused by the Young PRP over the Aged PRP to just one molecule. The anti-inflammatory effect of PRP is a known process mediated by several molecules such as IGF-1 and other growth factors, which are involved in the reduction in NF-κB activation, nitric oxide, cyclooxygenase, and tumor necrosis factor expression in the brain [5]. Several in vitro studies have indicated that this growth factor has an anti-inflammatory effect on astrocytes and microglia [35] and promotes the M2 phenotype in microglia [36]. This may contribute to tissue repair. TGFβ is also another factor that is implicated in the anti-inflammatory effect of PRP [37], and Young PRP presented higher levels than Aged PRP. In addition, as mentioned above, the more pro-inflammatory profile of Aged PRP due to its molecular composition may contribute to its lower anti-inflammatory capacity compared to Young PRP.

The main limitation of this study is inherent to in vitro studies since the results obtained are not always easy to translate to clinical outcomes. However, some of the cell models used in this work came directly from animal models, bringing the knowledge obtained closer to clinical application. Further preclinical and clinical studies are needed to consolidate the results obtained in the present study and to advance the field of CNS pathologic and potential therapeutic tools.

## 4. Materials and Methods

### 4.1. Platelet-Rich Plasma Preparation and Characterization

The institutional review board approved this study and informed consent was obtained from every patient from whom biological samples were extracted.

PRP was prepared from the peripheral blood of healthy donors: 12 donors aged 65–85 years old and eight younger donors 20–25 years old. First, 81 mL of venous blood were extracted from each donor in order to prepare the PRP and stored in 8-mL tubes containing 3.8% (wt/V) sodium citrate. Blood was centrifuged at 580× *g* for 8 min at room temperature. After centrifugation, the plasma fraction located above the sedimented red blood cells, was collected in a tube without including the buffy coat according to the protocol [38]. This obtaining process avoids the inclusion of white blood cells and reaches a moderate concentration of platelets (1 to 2 times the concentration of platelets compared with peripheral blood, depending on the platelet count and size as well as the hematocrit) and an absence of erythrocytes and leukocytes [39]. The plasma obtained from each patient was activated by adding CaCl_2_ (10% wt/vol), and the supernatant obtained after each plasma coagulation was mixed to get both Aged PRP and Young PRP.

Collected PRPs were assayed using a hematology analyzer to evaluate the erythrocytes, leukocytes, and platelets. Moreover, molecules and cytokines were analyzed by diverse ELISA kits and Proteome profiler human XL Cytokine array kit (R&D Systems, Minneapolis, MN, USA). Standards and samples were analyzed in duplicate according to the procedure specified in the kits.

### 4.2. In Vitro Assays

#### 4.2.1. Culture and Neuronal Differentiation of NT2 Cells

Human teratocarcinoma NTERA2-D1 cells (hereafter referred to as NT2) from the American Type Culture Collection (ATCC^®^, CRL-1973™, Manassas, VA, USA) were seeded on poly-D-lysine coated 12-mm glass coverslips pre-coated with 1:25 diluted Matrigel™ (BD Biosciences, Madrid, Spain), and maintained in a complete medium: Dulbecco’s Modified Eagle medium (DMEM^®^, ATCC 30-2002™), supplemented with 10% fetal bovine serum (FBS, Sigma-Aldrich, St. Louis, MO, USA) and antibiotics (100 U/mL penicillin and 100 μg/mL streptomycin, Gibco, Life Technologies S.A., Madrid, Spain). NT2 cells were routinely tested for mycoplasma contamination by PCR using the primers described by Uphoff et al. [40]. Positive and negative controls used for these tests were kindly donated by Dr. Uphoff (Leibniz-Institute DSMZ, Department of Human and Animal Cell Lines, Virus Diagnostics, Inhoffenstr. 7b, 38124 Braunschweig, Germany).

Cultures that had reached 70% confluence were either (i) induced to differentiate into neurons by treatment with the pyrimidine nucleoside analog cytosine β-D-arabinofuranoside (AraC) 20 µM for three days and analyzed for the effect of PRP on cell viability and morphology of these cells (ii) or deprived of FBS for 24 h to analyze the effect of PRP stimulation on 5-bromo-2′-deoxyuridine (BrdU) incorporation.

#### 4.2.2. PRP Treatment, Imaging, and Analysis of BrdU Incorporation in NT2 Progenitor Cells

NT2 cells deprived of FBS for 24 h were stimulated for an additional 24 h with either 10% FBS, 2% FBS, 2% Aged PRP or 2% Young PRP and then incubated with 10 µM 5-bromo-2′-deoxyuridine (BrdU; Thermo Fisher Scientific, Barcelona, Spain) for 4 h before being fixed for immunofluorescence labeling and analysis (see Appendix A, Section Appendix A.1). NT2 cells identically treated, but with no serum added, were used as the unstimulated control.

#### 4.2.3. PRP Treatment, Imaging, and Analysis of AraC-Differentiated NT2 Cells

After three days of differentiation, AraC 20 µM containing complete medium was replaced by a fresh complete medium supplemented or not with 2% Aged PRP or 2% Young PRP and maintained for three more days in culture. Cells were then fixed and processed for double immunofluorescence labeling as described in Appendix A (Sections Appendix A.2, Appendix A.3, Appendix A.4).

#### 4.2.4. Neuronal Primary Cultures

Primary neuron cultures were obtained from the cortical lobes of E18 Sprague–Dawley rat embryos according to previously described procedures [41]. Cells were resuspended in a B27 Neurobasal medium plus 10% FBS (Sigma, St. Louis, MO, USA) and seeded onto poly-l-ornithine-coated glass coverslips at 1 × 10^5^ cells per coverslip (12 mm in diameter) and 48-well plates at 1.5 × 10^5^ per well. One day later, the medium was replaced by serum-free, B27-supplementedneurobasal medium. The cultures were essentially free of astrocytes and microglia; they were maintained at 37 °C and 5% CO_2_. Cultures were used 8–10 days after plating.

#### 4.2.5. Toxicity Assay

A calcein assay was used for viability measurements and consequently to provide toxicity measurements as well. After the desired treatment, neurons were incubated with 1 µM calcein-AM (Invitrogen) for 30 min and data were acquired by a Synrgy-HT fluorimeter (Bio-Tek Instruments, Inc., Winooski, VT, USA).

#### 4.2.6. Western Blot for Synaptic Markers In Vitro

Lysates were prepared from rat culture primary neurons 7DIV using a modified RIPA buffer as previously described [42]. Protein concentrations were determined by the DC Protein Assay (Bio-Rad Laboratories, Hercules, CA, USA), and equal amounts of protein from each sample were analyzed by Bolt 4–12% Bis-Tris Plus SDS-PAGE (Thermo Fisher Scientific), followed by immunoblotting. The protein bands were detected with a ChemiDoc™ XRS Imaging System (Bio-Rad), and the band intensities are quantified by density using Quantity One^®^ (Bio-Rad) software. Synaptophysin and PSD-95 synaptic protein measurements were divided by the corresponding β-actin measurement for normalization. These normalized densities were then scaled so that the average value for the wild type was 100%.

#### 4.2.7. Immunofluorescence for Synaptic Markers In Vitro

Cocultured neurons at 10 DIV were fixed in 4% formaldehyde and 4% sucrose in PBS for 20 min, washed in PBS, permeabilized with 0.2% Triton-X for 5 min, and blocked in 3% BSA for 60 min. The cultures were incubated overnight at 4 °C with primary antibodies, anti-Synaptophysin and anti-Homer, in 2% NGS in PBS. Cultures were washed three times with PBST (0.05% Tween in PBS) and incubated with their respective secondary antibody for 1 h at RT. After appropriate washing, coverslips were mounted with Fluoromount-G (Southern Biotechnology Associates, Birmingham, AL, USA). In multiple-label experiments, channels were imaged sequentially to avoid bleedthrough. Immunofluorescence was examined by confocal microscopy using a Leica TCS STED CW SP8X confocal microscope (Leica, Wetzlar, Germany).

#### 4.2.8. Synaptic Marker Intensity and Puncta Analysis

Confocal images were acquired by a Leica TCS STED CW SP8X confocal microscope. One coverslip from each culture was analyzed, with 2–5 neurons per coverslip. From each neuron, 3–5 neuritic segments of 20 µm in length were selected from areas where either single process could be outlined. Images are thresholded for background removal. For synaptic marker analysis, the integrated intensity of each of the thresholded area was quantified using Fiji-ImageJ software. For synaptic puncta analysis, thresholded images were converted into binary images and regions of interest (ROI) were created. Each ROI within the segment was considered a punctum. The number of puncta within the segment is quantified. For colocalization of presynaptic synaptophysin and postsynaptic Homer, colocalizing ROIs between both channels were quantified within the segment using Fiji-ImageJ software. For each condition, individual segment measurements were averaged per embryo. The per-embryo averages were then used to calculate the group mean and standard deviation.

#### 4.2.9. Microglia Culture and Microglia Activation Assay

Primary mixed glial cultures were prepared from the cerebral cortex of neonatal rats (P0–P2) as previously described [43]. After 10–15 days in culture, microglia were isolated by mechanical shaking (400 rpm, 1 h) and purified by plating on noncoated bacterial grade Petri dishes (Sterilin), as previously described by Domercq et al. [43]. To analyze the impact of enriched plasma in microglia activation, cells were exposed (24 h) to lipopolysaccharide LPS (10 ng/mL) and IFNγ (20 ng/mL; Peprotech; London, UK) in the presence or absence of plasma or platelet-enriched plasma. To quantify microglia activation, we analyzed by immunocytochemistry the expression of inducible nitric oxide synthase (iNOS), a pro-inflammatory marker, and the expression of mannose receptor (MNR), an anti-inflammatory marker. The immunoreactivity of iNOS and MNR was calculated with ImageJ software (NIH) and normalized to the number of cells (eight fields per coverslip, from at least four different experiments performed in triplicate). Results were expressed as the change in fluorescence intensity relative to that seen in control cells or in LPS plus IFNγ-treated cells.

### 4.3. Statistical Analysis

Comparisons were performed by analysis of variance (ANOVA), and Student’s *t*-tests were used unless otherwise indicated. The normal distribution of samples was assessed by the Shapiro–Wilk test and the homogeneity of variance by the Levene test. In case the data did not fit the normal distribution or the variances were not homogeneous, nonparametric Kruskal–Wallis one-way ANOVA was applied. Data were considered statistically significant when the *p*-values were less than 0.05. Statistical analysis was performed with PASW Statistics 18.0 (SPSS^®^, Chicago, IL, USA) and GraphPad Prism (San Diego, CA, USA).

## 5. Conclusions

PRP properties can change dramatically depending on the age of the donor, with the PRP of aged donors having a more inflammatory profile. While both PRP act as modulating processes for CNS cells, PRP from young donors is more effective at reducing neural progenitor cell apoptosis, stabilizing neuronal synapses, and decreasing inflammation in the microglia. Therefore, the molecular composition of the PRP, conditioned by the age of the donors, affects the magnitude of the biological response.

## Figures and Tables

**Table 1:** Aged and Young PRP pools feature.

	*n*	Mean ± S.D. (Years)	Age Range (Years)
**Aged**	12	73.71 ± 5.00	(65–85)
**Young**	8	22.13 ± 1.81	(20–25)

**Table 2:** Summary of characteristics for PRP.

	Aged PRP	Young PRP
**1. PRP Preparation**		
Initial blood volume	81 mL per subject (9 mL per tube)	81 mL per subject (9 mL per tube)
Anticoagulant	Sodium citrate 3.8% (wt/V)	Sodium citrate 3.8% (wt/V)
System	Close	Close
Centrifugation	Yes	Yes
*number*	*1*	*1*
*Speed*	*580 g—8 min*	*580 g—8 min*
Final PRP volume	18 mL per subject	18 mL per subject
**2. PRP Characteristics**		
PRP Type	12-00-11	12-00-11
MPV	10.4 ± 0.5 fL	9.6 ± 0.5 fL
Red Blood Cells	<0.01 × 10^6^/µL	<0.01 × 10^6^/µL
White Blood Cells	<0.05 × 10^6^/µL	<0.05 × 10^6^/µL
*Neutrophils*	---	---
*Lymphocytes*	---	---
*Monocytes*	---	---
*Eosinophils*	---	---
*Basophils*	---	---
Activation	CaCl_2_ (10% wt/vol)	CaCl_2_ (10% wt/vol)
**3. Application Characteristics**		
Dose	2%	2%
Direct/Indirect	Direct	Direct
Cell line	CNS cells	CNS cells
**4. Other Remarkable PRP and Study Features**		
	The product added to the cell cultures was the platelet lysate obtained after activation of PRP with calcium chloride (10%)	

**Table 3:** Concentrations of growth factors and cytokines in Aged and Young groups.

Growth Factors and Cytokines	Aged PRP Mean ± S.D. (pg/mL)	Young PRP Mean ± S.D. (pg/mL)	*p*
CCL-11	72.51 ± 19.00	46.75 ± 13.56	0.022 *
GDF-11	0	169.04 ± 6.25	<0.001 ***
G-CSF	209.91 ± 88.92	143.82 ± 69.10	0.181
HGF	1101.45 ± 44.09	699.33 ± 5.66	0.001 **
IGF	53,036.72 ± 9254.97	106,320.62 ± 10,470.11	<0.001 ***
PDGF	2834.75 ± 427.59	3908.59 ± 705.04	0.016 *
TGF-β	34,732.53 ± 3657.98	42,332.98 ± 4958.67	0.025 *
TNF-α	17.55 ± 0.98	18.10 ± 1.75	0.600

* p < 0.05, ** *p* < 0.01, *** *p* < 0.001.

**Table 4:** Cytokine values.

Number	Coordinate	Analyte	Aged PRP Group (Mean ± S.D.) (IOD)	Young PRP Group (Mean ± S.D.) (IOD)	*p* Value	Number	Coordinate	Analyte	Aged PRP Group (Mean ± S.D.) (IOD)	Young PRP Group (Mean ± S.D.) (IOD)	*p* Value
1	A3, A4	Adiponectin	3989.26 ± 304.47	4154.84 ± 80.55	0.333	27	E3, E4	IL-5	364.37 ± 230.30	286.47 ± 77.85	0.545
2	A5, A6	Apolipoprotein A-I	4524.69 ± 269.84	4493.79 ± 272.99	0.877	28	E23, E24	IL-18 Bpa	3355.77 ± 75.21	2785.41 ± 499.27	0.064
3	A7, A8	Angiogenin	4835.93 ± 61.23	4682.65 ± 364.25	0.438	29	G1, G2	Leptin	1832.24 ± 397.51	468.69 ± 3.88	<0.001 *
4	A9, A10	Angiopoietin-1	1705.54 ± 134.90	995.56 ± 128.65	<0.001 *	30	G5, G6	Lipocalin-2	3055.21 ± 136.35	1501.53 ± 60.29	<0.001 *
5	A11, A12	Angiopoietin-2	397.67 ± 46.55	203.83 ± 81.25	0.006 *	31	G13, G14	MIF	752.34 ± 36.06	344.14 ± 52.74	<0.001 *
6	A13, A14	BAFF	1199.89 ± 75.92	833.94 ± 289.97	0.050	32	G21, G22	MIP-3β	239.42 ± 93.85	81.19 ± 37.61	0.020 *
7	A17, A18	C5/C5a	2596.91 ± 336.86	1945.71 ± 203.49	0.016 *	33	G23, G24	MMP-9	1409.28 ± 229.17	871.01 ± 102.66	0.005 *
8	A19, A20	CD14	1803.53 ± 217.21	1471.18 ± 329.11	0.142	34	H1, H2	Myeloperoxidase	439.91 ± 177.34	322.67 ± 45.61	0.247
9	A21, A22	CD30	124.93 ± 37.07	101.39 ± 118.70	0.718	35	H3, H4	Osteopontin	2872.29 ± 534.43	3260.3 ± 129.74	0.088
10	B3, B4	CD40 ligand	585.14 ± 117.44	735.12 ± 44.84	0.054	36	H9, H10	Pentraxin 3	407.46 ± 93.16	293.89 ± 69.21	0.098
11	B5, B6	Chitinase 3-like 1	3953.46 ± 211.37	3015.32 ± 54.49	<0.001 *	37	H11, H12	PF4	3927.03 ± 350.75	3697.05 ± 114.58	0.263
12	B7, B8	Complement Factor D	2556.74 ± 118.44	2444.56 ± 105.74	0.207	38	H15, H16	RANTES	2685.81 ± 85.30	2257.70 ± 98.68	0.001 *
13	B9, B10	C-Reactive Protein	4920.45 ± 152.84	4531.85 ± 273.50	0.047 *	39	H17, H18	RBP-4	4169.65 ± 662.79	3547.42 ± 242.99	0.128
14	B11, B12	Cripto-1	2199.56 ± 296.10	1683.65 ± 108.46	0.016 *	40	H21, H22	Resistin	1452.82 ± 110.10	781.19 ± 43.31	<0.001 *
15	B15, B16	Dkk-1	369.85 ± 32.40	259.23 ± 104.68	0.090	41	H23, H24	SDF-1α	540.59 ± 146.77	252.61 ± 35.56	0.008 *
16	B17, B18	DPPIV	4820.59 ± 337.84	4297.65 ± 253.27	0.047 *	42	I1, I2	Serpin E1	5621.19 ± 241.19	5263.41 ± 297.57	0.110
17	B21, B22	Emmprin	1067.68 ± 137.51	622.89 ± 376.77	0.068	43	I3, I4	SHBG	4755.72 ± 249.73	4513.15 ± 206.17	0.184
18	C3, C4	ENA-78	2057.37 ± 66.20	2209.68 ± 150.08	0.112	44	I5, I6	ST2	1107.68 ± 81.92	746.5 ± 144.46	0.004 *
19	C5, C6	Endoglin	3398.28 ± 152.91	3700.11 ± 98.83	0.016 *	45	I7, I8	TARC	905.01 ± 42.82	603.39 ± 169.32	0.013 *
20	C13, C14	FGF-19	305.75 ± 42.74	142.15 ± 29.98	<0.001 *	46	I9, I10	TFF3	1112.75 ± 73.10	1314.63 ± 179.57	0.082
21	C15, C16	Flt-3 Ligand	139.46 ± 42.29	54.35 ± 8.87	0.007 *	47	I11, I12	TfR	852.16 ± 73.98	571.47 ± 85.82	0.002 *
22	C19, C20	GDF-15	1166.25 ± 133.84	230.15 ± 153.84	<0.001 *	48	I15, I16	Thrombospondin1	2055.38 ± 123.63	1473.03 ± 297.34	0.025 *
23	D3, D4	Growth Hormone	652.69 ± 130.68	1380.33 ± 187.64	<0.001 *	49	I19, I20	uPAR	700.47 ± 33.23	189.58 ± 56.00	0.001 *
24	D7, D8	ICAM-1	978.39 ± 193.86	733.12 ± 160.05	0.098	50	J7, J8	CD31	3444.59 ± 256.88	3132.6 ± 49.38	0.054
25	D11, D12	IGFBP-2	3227.19 ± 71.56	2700.56 ± 82.07	<0.001 *	51	J9, J10	TIM-3	2973.56 ± 122.94	2075.5 ± 114.34	<0.001 *
26	D13, D14	IGFBP-3	1146.28 ± 155.31	640.54 ± 138.50	0.002 *	52	J11, J12	VCAM-1	4394.29 ± 100.97	4041.8 ± 116.81	0.03

* *p* < 0.05.

**Figure A1:**
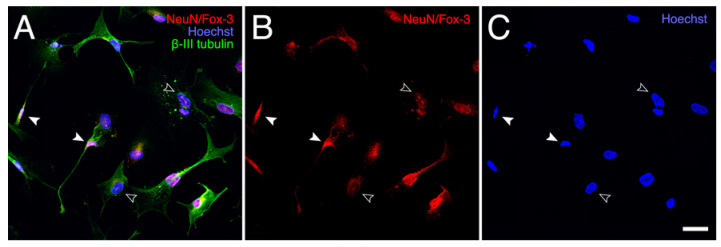
Representative image of stained AraC-treated NT2 cells used for morphometric analysis. Double immunofluorescence labeling using antibodies against the neuronal and postmitotic neuron markers β-III tubulin (green) and NeuN/Fox-3 (red), respectively. Cell nuclei were counterstained with the chromatin marker Hoechst 33342 (blue) (**A**). Staining corresponds to the NeuN/Fox-3 (**B**) and Hoechst (**C**) channels. Filled and empty arrowheads depict examples of neuronal and non-neuronal cells, respectively. Scale bar: 50 µm.

**Figure A2:**
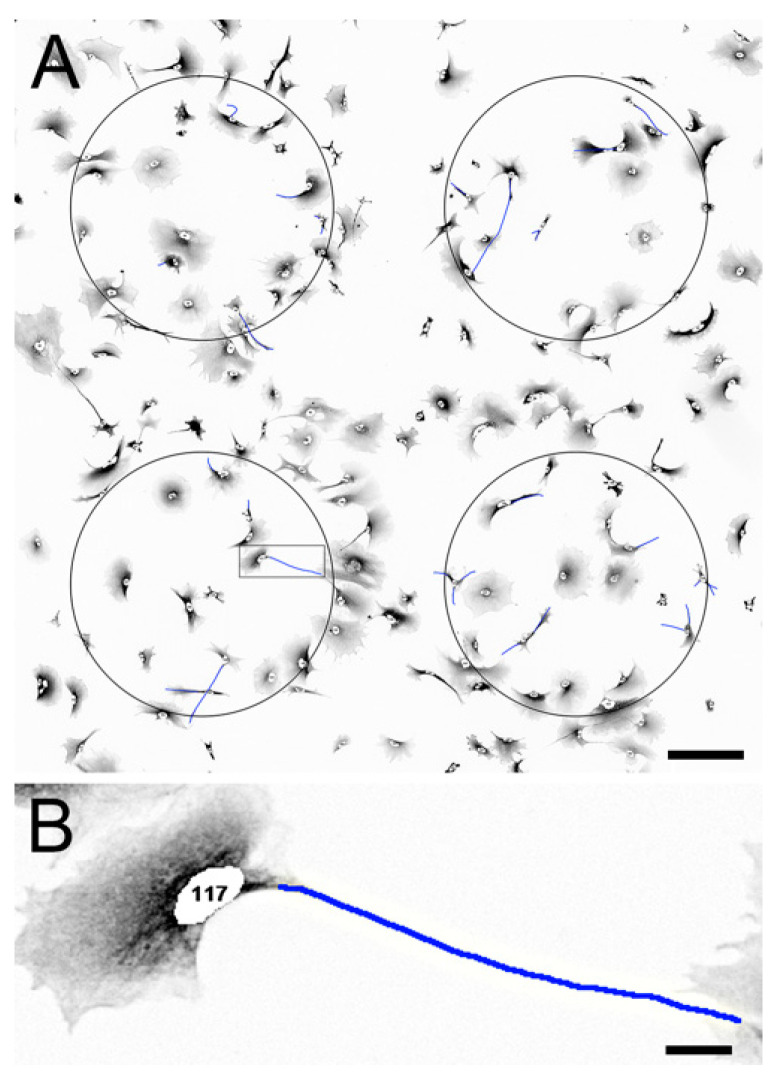
Grayscale image of the signal corresponding to the β-III tubulin channel of β-III tubulin and NeuN/Fox-3 doubly immunolabeled cells. Circles depict sampling areas (ROI). Traced neurites located with the NeuronJ plugin of ImageJ software are depicted in blue (**A**). (**B**) Detail of the framed area in (**A**). Scale bars: 200 µm in (**A**); 20 µm in (**B**).

## Data Availability

The data presented in this study are available within the article.

## Abbreviations

AraC	Pyrimidine nucleoside analogue cytosine β-D-arabinofuranoside
BrdU	5-bromo-2′-deoxyuridine
CNS	Central nervous system
FBS	Fetal bovine serum
LPS	Lipopolysaccharide
NT-2	Human teratocarcinoma NTERA2-D1 cells
PRP	Platelet-rich plasma
CCL-11	C-C motif chemokine 11
GDF-11	Growth differentiation factor
G-CSF	Granulocyte colony-stimulating factor
HGF	Hepatocyte growth factor
IGF	Insulin-like growth factor
PDGF	Platelet-derived growth factor
TGF-β	Transforming growth factor-β
TNF-α	Tumor necrosis factor

## Appendix A

### Appendix A.1. BrdU Immunofluorescence Labeling

Fixed NT2 cells intended for BrdU immunofluorescence labeling were permeabilized with PBS containing 0.01% TX-100 for 10 min and then treated with HCl 1 N for 30 min at 37 °C, followed by neutralization with sodium borate 0.1 M at pH 8.5 for 10 min at 20–25 °C. Cells were then washed thoroughly with wash buffer before proceeding with BrdU immunofluorescence, which was performed as described above (except that the blocking buffer did not contain normal donkey serum), with the primary antibody rat anti-BrdU monoclonal antibody (clone BU1/75 -ICR-; Novus Biologicals^®^, Madrid, Spain) and the secondary antibody Alexa Fluor 568 Goat anti-Rat IgG (A-11077, Invitrogen SA, Barcelona, Spain), diluted to 1:2000 and 1.200, respectively.

Microscope images were acquired using the equipment and settings described above, except that only 43 HE Cy3 shift-free (Ex 550/25, Em 605/70) and 49 DAPI (Ex G 365/Em 445/50) bandpass filters were used for Alexa Fluor 568 (BrdU staining) and Hoechst’s staining, respectively. Fifteen ROI of 0.15 mm^2^ per coverslip were randomly selected using the Carl Zeiss “Mark&Find” module of the Axio Vision Rel. 4.8 acquisition software and acquired with identical exposure and lighting conditions for quantitative analysis of BrdU incorporation. Using Fiji-ImageJ software, nuclei displaying BrdU staining above threshold level were scored as positive. The number of BrdU-positive and the total number of nuclei (stained with Hoechst) were counted using the Cell Counter plugin of ImageJ. The BrdU Incorporation Index was calculated as the percentage of BrdU-positive nuclei relative to all Hoechst-stained nuclei found in two coverslips per experimental condition (30 ROI) from five independent experiments (*n* = 5). Statistical analysis of the data was performed by one-way ANOVA and Bonferroni post hoc test, using GraphPad Prism 5.0 software. All data were expressed as the average of the values ± SEM. For illustration, images exported as TIFF files were compiled and labeled using Adobe Photoshop CS3 (San Jose, CA, USA).

### Appendix A.2. Immunofluorescence in NT2 Cells

Cells were fixed with 4% paraformaldehyde in 0.1 M phosphate buffer, pH 7.4 (PB) for 5 min at 20–25 °C and washed three times with 0.1 M phosphate-buffered saline, pH 7.4 (PBS) before immunofluorescence labeling.

### Appendix A.3. Double Immunofluorescence Staining of AraC-Differentiated NT2 Cells

Fixed AraC-differentiated cells intended for cell viability and morphology analysis were washed briefly with a wash buffer, consisting of PBS containing 0.22% gelatin (Panreac, Barcelona, Spain), and incubated for 1 h with a permeabilizing blocking buffer (washing buffer with 0.05% saponin, Sigma-Aldrich; 1% serum albumin bovine BSA, Sigma-Aldrich; 1% normal goat serum; and 1% normal donkey serum). Thereafter, cells were incubated at 4 °C overnight with an affinity-purified chicken serum against the neurite marker β-III-tubulin (ab41489, Abcam, Cambridge, UK) combined with a mouse monoclonal antibody against the marker of postmitotic neuronal nuclei NeuN/Fox-3, diluted to 1:1000 and 1:200, respectively, in blocking buffer. After three washes of 10 min with a washing buffer, cells were incubated for 1 h at 20–25 °C with secondary antibodies Dylight 488 donkey anti-chicken IgG (703-486-155, Jackson InmunoResearch, West Grove, PA, USA) and Alexa Fluor 568 goat anti-mouse IgG (A-11031, Invitrogen SA, Spain), both diluted to 1:400 in blocking buffer. Finally, cells were washed twice for 10 min with washing buffer and counterstained with Hoechst 33342 (Sigma-Aldrich; 0.1 μg/mL in washing buffer). After two additional washes of 10 min in PBS, coverslips containing immunolabeled cells were mounted onto glass slides using homemade Mowiol (Calbiochem, Madrid, Spain) mounting medium containing antifade reagent 1,4-phenylenediamine dihydrochloride (Sigma-Aldrich).

### Appendix A.4. Imaging and Analysis of AraC-Differentiated NT2 Cells

Images of β-III-tubulin and NeuN/Fox-3 doubly labeled cells were obtained using a Carl Zeiss Axio Observer Z1 epifluorescence microscope, equipped with an XYZ motorized stage, using an HXP120C metal halide lamp as a light source (all from Carl Zeiss MicroImaging, Inc., Gottingen, Germany). Cells were imaged with a 20× Plan-Apochromat (NA 0.8; pixel size 0.322 × 0.322 mm^2^) from Carl Zeiss MicroImaging, Inc. Bandpass filters from Carl Zeiss MicroImaging, Inc were 43 HE Cy3 shift-free (Ex 550/25, Em 605/70) for Alexa Fluor 568 (β-III-tubulin staining), 38 HE eGFP (Ex 470/40, Em 525/50) for Dylight 488 (NeuN/Fox-3 staining) and 49 DAPI (Ex G 365/Em 445/50; Hoechst’s staining) (Figure A1). The three channels were captured independently in grayscale, setting an identical exposure time for all the captures of each channel. After placing the coverslip in the center of the objective, several digital images were acquired with focus adjustment at predefined positions using the Carl Zeiss Mosaic module of the Axio Vision Rel. 4.8 acquisition software and a high-resolution monochrome camera AxioCam MRm (1388 × 1040 pixels) at a 16-bit depth. From each preparation, a mosaic with a total area of 7.84 µm^2^ was created, where four circular sampling areas of 0.385 µm^2^ each were delimited (3.08 µm^2^ per preparation; Figure 2A). Finally, mosaic mages were saved in the TIFF format for subsequent analysis using ImageJ software (NIH, Bethesda, MD, USA). All cells whose nuclei fell within the sampling areas (regions of interest; ROI) were analyzed. In total, three independent experiments were carried out, analyzing every two replicates (eight ROI) per condition. As parameters related to cell viability and cell phenotype, the density of neuronal (NT2N) and non-neuronal cells was calculated, as well as the percentage of NT2N cells with respect to the total population. As for morphometric parameters, the mean area of the cell nucleus was calculated in the neuronal and non-neuronal phenotypes, as well as the mean number of neurites and the total length of the neuritic tree of the NT2N cells.

First, cells were categorized into NT2N (neuronal) and non-neuronal using a simple algorithm based on two discrimination criteria: on the one hand, the area of the cell nucleus, which is significantly smaller in the NT2N cells (Figure 1; González-Burguera et al., 2016a, b); and, on the other hand, the intensity of NeuN/Fox-3 immunostaining within the cell nucleus, which is stronger in NT2N cells. In each ROI, the perimeter of nuclei was established on images corresponding to the chromatin marker Hoechst 33342 by generating a binary image using the threshold module of Fiji-ImageJ software. Then, the area of nuclei and the mean optical density of NeuN/Fox-3 staining within nuclear perimeters were measured (ROI edges excluded) using Fiji-ImageJ. Data obtained for both parameters in each ROI were normalized by calculating the quotient between the value obtained in each nucleus and the highest value obtained. Thus, a normalized area value (AN = A/Amax) and a normalized NeuN/Fox-3 optical density value (ODN = OD/ODmax) were obtained for each cell nucleus. Subsequently, “neuronality” was assessed using the following equation: Neuronality = (1 − AN) × ODN. Neuronal cells were considered those that fulfilled the following two criteria: (i) displayed a neuronality value above the average of the cell population in a given ROI, and (ii) possessed at least one neurite. For the analysis of the length and number of neurites, the grayscale images corresponding to the β-III tubulin signal were inverted and overlapped with the binary image corresponding to cell nuclei (Figure A2), thus facilitating the visualization of the full cell morphology. On these images, the length of the neurites was measured using the NeuronJ plugin in Fiji-ImageJ. The cell density values and the percentages of NT2N and non-neuronal neuronal cells were calculated from the data obtained from three independent tests (*n* = 3) with two replicates per test and four sampling areas per replicate. The area of the cell nucleus was measured in a total of 342 cells classified as NT2N neuronal (112 FBS, 112 Aged PRP, and 118 Young PRP) and 488 cells classified as non-neuronal (112 FBS, 178 Aged PRP, and 198 Young PRP) from all the ROIs of the three independent tests. The length and number of neurites were analyzed in the 342 cells identified as neuronal NT2N mentioned above. Statistical analysis of the data was performed by one-way or two-way ANOVA and Bonferroni post hoc test, using GraphPad Prism 5.0 software. All data were expressed as the average of the values ± SEM. For illustration, images exported as TIFF files were compiled and labeled using Adobe Photoshop CS3 (San Jose, CA, USA).
